# Cholesterol 25‐Hydroxylase Protects Against Diabetic Kidney Disease by Regulating ADP Ribosylation Factor 4

**DOI:** 10.1002/advs.202309642

**Published:** 2024-05-30

**Authors:** Lu Zhang, Zhengying Fang, Qingqing Zhu, Shumin Yang, Jia Fu, Zeguo Sun, Geming Lu, Chengguo Wei, Zhi Zhang, Kyung Lee, Yifei Zhong, Ruijie Liu, John Cijiang He

**Affiliations:** ^1^ Department of Medicine Division of Nephrology Icahn School of Medicine at Mount Sinai New York NY Box 1243 USA; ^2^ Division of Endocrinology Diabetes and Bone Diseases Icahn School of Medicine at Mount Sinai Diabetes Obesity and Metabolism Institute One Gustave L. Levy Place New York NY 10029 USA; ^3^ Département de Génétique Laboratoire national de santé Dudelange Dudelange L‐3555 Luxembourg; ^4^ Division of Nephrology Longhua Hospital Shanghai University of Traditional Chinese Medicine 725 South Wanping Road Shanghai 200032 China; ^5^ Renal Section James J Peter Veterans Administration Medical Center Bronx NY 10468 USA

**Keywords:** diabetic kidney diseases, glomerular endothelial cells, golgi apparatus

## Abstract

Cholesterol 25‐hydroxylase (CH25H), an enzyme involved in cholesterol metabolism, regulates inflammatory responses and lipid metabolism. However, its role in kidney disease is not known.  The author found that CH25H transcript is expressed mostly in glomerular and peritubular endothelial cells and that its expression increased in human and mouse diabetic kidneys.  Global deletion of *Ch25h* in Lepr^db/db^ mice aggravated diabetic kidney disease (DKD), which is associated with increased endothelial cell apoptosis. Treatment of 25‐hydroxycholesterol (25‐HC), the product of CH25H, alleviated kidney injury in Lepr^db/db^ mice. Mechanistically, 25‐HC binds to GTP‐binding protein ADP‐ribosylation factor 4 (ARF4), an essential protein required for maintaining protein transport in the Golgi apparatus. Interestingly, ARF4's GTPase‐activating protein ASAP1 is also predominantly expressed in endothelial cells and its expression increased in DKD. Suppression of ARF4 activity by deleting ARF4 or overexpressing ASAP1 results in endothelial cell death. These results indicate that 25‐HC binds ARF4 to inhibit its interaction with ASAP1, and thereby resulting in enhanced ARF4 activity to confer renoprotection. Therefore, treatment of 25‐HC improves kidney injury in DKD in part by restoring ARF4 activity to maintain endothelial cell survival. This study provides a novel mechanism and a potential new therapy for DKD.

## Graphical abstract

This study shows that cholesterol 25‐hydroxylase (CH25H), a key enzyme in cholesterol metabolism, is expressed in kidney endothelial cells and protects against diabetic kidney disease. Its product, 25‐HC, binds to and enhances the activity of GTP‐binding protein ADP‐ribosylation factor 4 (ARF4), an essential protein required for Golgi trafficking and homeostasis, to promote endothelial cell survival and slow disease progression.

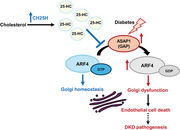

## 1 Introduction

Diabetic kidney disease (DKD) is a leading cause of chronic kidney disease worldwide.^[^ ^1^ ^]^ Renin–angiotensin system inhibitors, sodium‐glucose cotransporter‐2 inhibitors, and mineralocorticoid receptor antagonists are the current standard therapies for DKD.^[^ ^2^ ^]^ However, with these therapies, many DKD patients still progress to end‐stage kidney disease,^[^ ^3^ ^]^ indicating an unmet need for therapies. Therefore, a better understanding of the molecular mechanisms mediating the DKD progression are still critically needed for the identification of potential new therapeutic targets.

Glomerular endothelial cell (GEC) dysfunction has long been implicated in the pathophysiology of DKD.^[^ ^4^ ^]^ However, the molecular changes that occur specifically in GECs in early DKD are poorly understood. To dissect the mechanisms of GEC injury in early DKD in an unbiased way, we previously conducted a transcriptomic analysis of sorted GECs from diabetic mice lacking endothelial nitric oxide synthase,^[^ ^5^ ^]^ as well as single‐cell transcriptomic analysis of glomerular cells from the same mouse model.^[^ ^6^ ^]^ These studies identified several important genes that are highly regulated in GECs in early DKD, among which were *Lrg1*,^[^ ^7^ ^]^ *Gpr56*,^[^ ^7^, ^8^ ^]^ and *Ch25h*.

CH25H is an enzyme that catalyzes cholesterol to generate 25‐hydroxycholesterol (25‐HC).^[^ ^9^ ^]^ CH25H and 25‐HC are involved in a wide range of biological processes, including anti‐viral modulation, inflammatory regulation, and lipid metabolism.^[^ ^10^ ^]^ However, recent studies have reported seemingly opposing roles of CH25H in varying disease contexts. For instance, CH25H protects against endothelial cell injury in atherosclerosis,^[^ ^11^ ^]^ while 25‐HC can aggravate endothelial injury in acute lung injury by inducing adhesion molecules and exacerbating vascular leakage.^[^ ^12^ ^]^ These reports suggest that CH25H and its metabolite 25‐HC may have tissue‐specific effects under different disease conditions.

CH25H is highly expressed in kidney cells,^[^ ^13^ ^]^ but its function in kidney cell biology or pathology has not been previously examined. Using genetic and pharmacological approaches, our study now demonstrates that 25‐HC, the enzymatic product of CH25H, provides renoprotection in diabetic kidneys by binding to and enhancing the activation of ADP ribosylation factor‐4 (ARF4). ARF proteins are regulatory GTPases that control a variety of cellular functions,^[^ ^14^ ^]^ and activated ARF4 is essential for maintaining the normal transportation function of the Golgi apparatus.^[^ ^15^ ^]^ We found that ARF4 activity is reduced in DKD due to the increased expression of its negative regulator GTPase‐activating protein, ASAP1. 25‐HC enhanced ARF4 activity by interrupting the interaction of ARF4 with ASAP1, thereby mitigating endothelial cell injury in diabetic kidneys.

## 2 Results

### 2.1 Expression of CH25H is increased in GECs of mouse and human iabetic Kidneys

**Figure 1:**
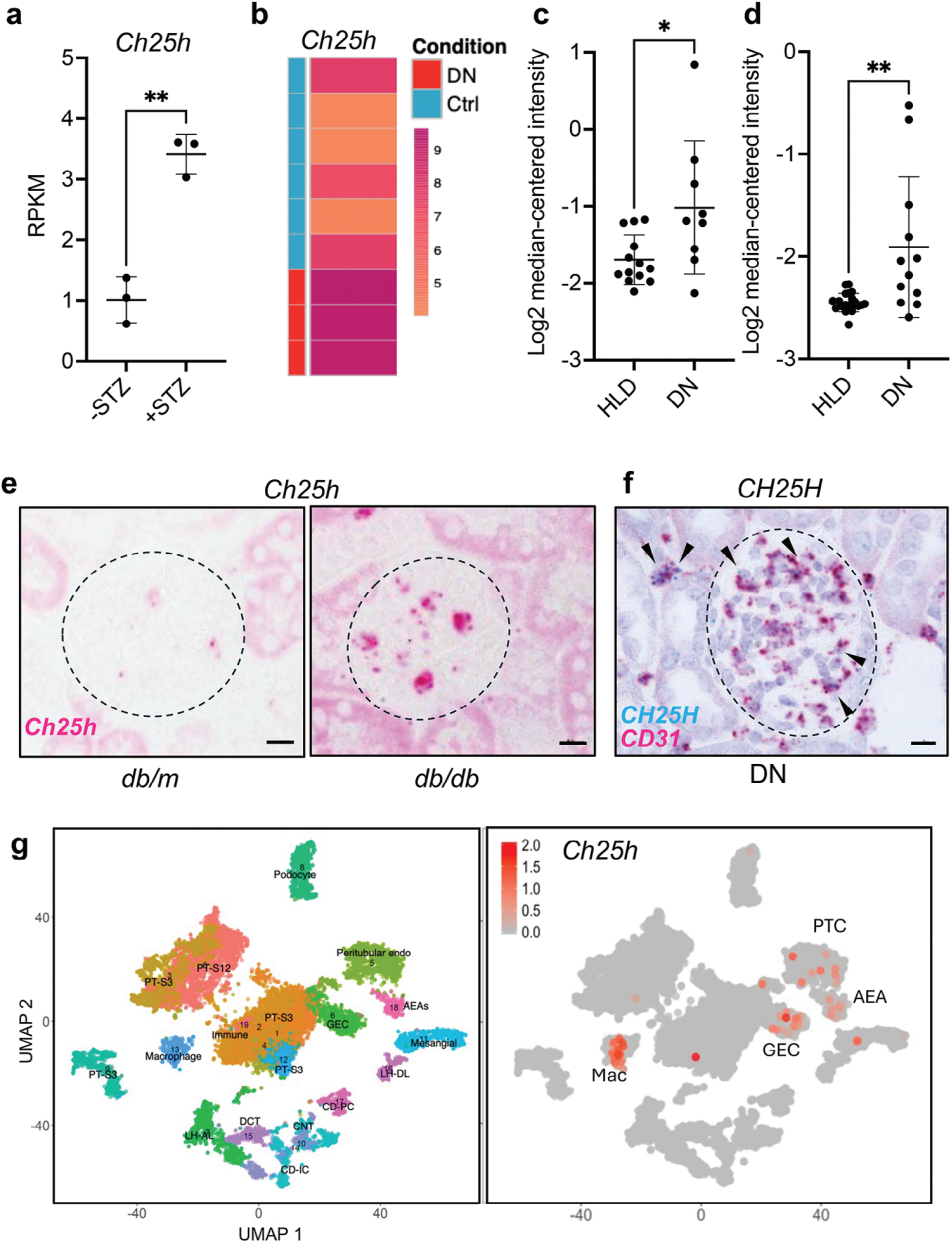
CH25H expression is increased in glomeruli of mouse and human diabetic kidneys and is predominantly expressed in endothelial cells. a) RNA‐sequencing data of *Ch25h* expression in isolated GECs of control (−STZ) or streptozotocin (+STZ)‐induced diabetic mice from Fu et al., 2018 (*n* = 3 samples, each sample consisted of pooled GECs from 4 mice). RPKM reads per kilobase per million mapped reads. b) *Ch25h* expression in diabetic mouse glomerular endothelial from the dataset by Brunskill et al.,2010. c,d) CH25H mRNA levels in human glomeruli isolated from DKD patients (DN) compared to healthy controls (HLD) in Woroniecka dataset (c) and Ju CKD dataset (d), accessed from Nephroseq database. e) In situ hybridization of *Ch25h* mRNA in kidneys of diabetic *db/db* mice and control *db/m* mice. Scale bar = 10 µm. Glomeruli are outlined. f) In situ hybridization of CH25H mRNA (blue) co‐localized with CD31 mRNA (pink) in human DN kidney. Scale bar = 10 µm. Arrowheads show examples of co‐localization. g) Single‐cell RNA‐sequencing analysis of *Ch25h* in mouse kidneys, which is predominantly expressed in PTC, GEC, and macrophages (Mac). *P < 0.05, and **P < 0.01 by *t*‐test.

Our recent transcriptomic profiling of isolated GECs from control and streptozotocin (STZ)‐induced diabetic mice ^[^ ^5^ ^]^ confirmed that *Ch25h* is one of the highly upregulated genes in diabetic mouse GECs (**Figure** **1**a). Consistent with this, the transcriptomic data of isolated GECs by Brunskill et al.^[^ ^16^ ^]^ also showed an increased *Ch25h* expression in the diabetic mice (Figure 1b). Increased CH25H level was similarly observed in isolated glomeruli of DKD patients in the Nephroseq database (Woroniecka DKD and Ju CKD datasets, *nephroseq.org*) (Figure 1c,d). In situ hybridization confirmed a marked increase in glomerular *Ch25h* mRNA expression Lepr^db/db^ mice as compared to the control Lepr^db/m^ mice (Figure 1e) and in CH25H mRNA expression in human diabetic kidneys (Figure 1f), which co‐localized predominantly with *CD31*‐expressing cells. Notably, CH25H expression was not limited to GECs, but also present in peritubular capillary endothelial cells (Figure 1f). Analysis of recent scRNA‐seq of mouse kidneys data further confirmed the endothelial cell localization of *Ch25h* in the mouse kidneys (Figure 1g). Together, these data indicate that CH25H expression is increased in endothelial cells in diabetic kidneys.

### 2.2 Genetic Deletion of Ch25h aggravates Albuminuria and Glomerulopathy in Diabetic Mice

**Figure 2:**
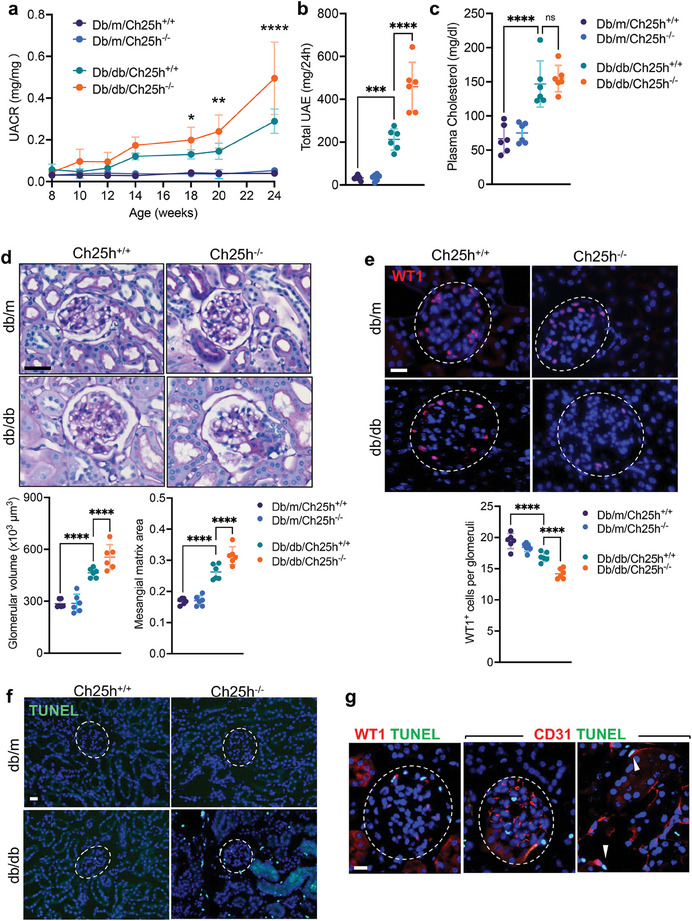
*Ch25h* ablation aggravates diabetes‐induced glomerulopathy, kidney injury, podocyte loss, and kidney cell death.  a) UACR. b) Total 24‐hour UAE at 24 weeks of age. c) Plasma cholesterol levels at 24 weeks of age. d) Representative images of periodic acid–Schiff‐stained kidneys (top), and quantification of glomerular volume and mesangial matrix fraction per mouse (bottom, 20–30 glomeruli counted per mouse, *n* = 6 mice per group). Scale bar = 20 µm. e) Representative images of WT1 immunofluorescence (top) and quantification of WT1‐positive podocytes per mouse (bottom, 20–30 glomeruli counted per mouse, *n* = 6 mice per group). Glomeruli are outlined. Scale bar, 20 µm. f) Representative images of TUNEL staining. Scale bar = 20 µm. g) Representative images of TUNEL‐positive cells in the diabetic *Ch25h* ^−/−^ mouse kidneys, co‐stained with podocyte marker WT1 or endothelial cell marker CD31. Glomerular regions (left and middle) and tubulointerstitial region (right panel) are shown. White arrowheads highlight examples of TUNEL+ peritubular capillary endothelial cells (right panel). Scale bar, 20 µm. *n* = 6 mice per group, *P < 0.05, **P < 0.01, ***P < 0.001, ****P < 0.0001 by 2‐way ANOVA.

To determine the role of CH25H in DKD, we crossed the type 2 diabetic Lepr^db/db^ mice with global CH25H knockout (*Ch25h* ^−/−^) mice. *Ch25h* ^−/−^ mice were grossly normal, as reported previously.^[^ ^12^, ^17^ ^]^ Non‐diabetic Lepr^db/m^/*Ch25h* ^+/+^ and Lepr^db/m^/*Ch25h* ^−/‐^ mice served as controls. All mice were euthanized at 24 weeks of age. Diabetes‐induced albuminuria, assessed by the urinary albumin–creatinine ratio (UACR) of bi‐weekly collected spot urine samples between 8 to 24 weeks of age (**Figure** **2**a) and total 24‐hour urine albumin excretion (UAE) at 24 weeks of age (Figure 2b) were markedly higher in Lepr^db/db^/Ch25h^−/−^ mice in comparison to Lepr^db/db^/Ch25h^+/+^ mice. However, no significant change in the total plasma cholesterol levels was observed between the diabetic mouse groups (Figure 2c). Histologic analysis of periodic acid‐Schiff (PAS)‐stained kidneys worsened diabetic glomerular hypertrophy and mesangial expansion in Lepr^db/db^/Ch25h^−/−^ mice compared to Lepr^db/db^/Ch25h^+/+^ mice (Figure 2d). Diabetes‐induced podocyte loss, as ascertained by podocyte marker WT‐1, was exacerbated in diabetic Ch25h^−/−^ mice compared to diabetic Ch25h^+/+^ mice (Figure 2e). Terminal deoxynucleotidyl transferase dUTP nick end labeling (TUNEL) of mouse kidneys showed increased kidney cell death in Lepr^db/db^/Ch25h^−/−^ mice (Figure 2f). To reveal which cell types undergoing cell death, we performed co‐staining of TUNEL with podocyte marker (WT1) or endothelial cell marker (CD31) of Lepr^db/db^/Ch25h^−/−^ mouse kidneys. TUNEL‐positive cells co‐localized mostly with CD31‐positive cells in both glomerular and tubulointerstitial compartments (Figure 2g), suggesting that GECs and peritubular capillary (PTC) cells are the major cell types undergoing cell death in Lepr^db/db^/*Ch25h* ^−/−^ mice kidneys. Some WT‐1 podocytes were also TUNEL‐positive (Figure 2g), suggesting that podocyte apoptosis also occurred possibly via GEC‐podocyte crosstalk. Together, these data indicated that genetic ablation of *Ch25h* resulted in marked aggravation of diabetic glomerulopathy likely via inducing endothelial cell injury.

### 2.3 25‐HC Treatment Attenuates diabetes‐induced Albuminuria and Glomerulopathy

**Figure 3:**
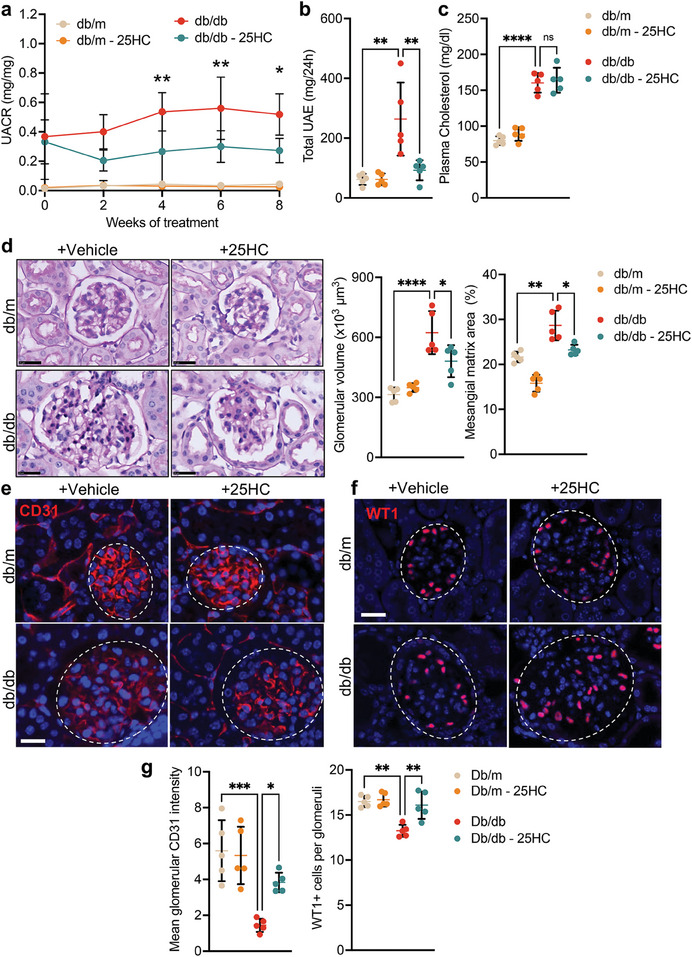
25‐HC treatment protects against diabetic glomerulopathy. a) UACR over time with 25‐HC treatment (*n* = 5 mice per group). b) 24‐hour UAE at 8 weeks post 25‐HC treatment (*n* = 5 mice per group). c) Plasma total cholesterol at 8 weeks post 25‐HC treatment (*n* = 5 mice per group). d) Representative images of PAS‐stained kidneys and quantification of glomerular volume and mesangial matrix area (%) per mouse (20–30 gloms counted per mouse, *n* = 5 mice). Scale bar = 25 µm. e) Representative images of CD31 immunofluorescence. Glomeruli are outlined with white dotted lines. Scale bar = 20 µm.  f) Representative images of WT1 immunofluorescence. Scale bar = 20 µm.  g) Quantification of the mean glomerular intensity of CD31 and WT1 per mouse. (*n *= 5 mice per group, 20–30 glomeruli scored per mouse). *P < 0.05, **P < 0.01, ***P < 0.001, ****P < 0.0001 by 2‐way ANOVA. ns, non‐significant.

As 25‐HC is the enzymatic product of CH25H, we next examined whether supplementing diabetic mice with 25‐HC would attenuate diabetic albuminuria and glomerulopathy in vivo. Lepr^db/m^ and Lepr^db/db^ mice were treated with a control vehicle or 25‐HC (0.16 mg kg^−1 ^ body weight), starting from 10 weeks of age for 8 weeks. Induction of diabetes was confirmed by measurement of blood glucose. Body weight and blood glucose levels are summarized in Table S1 (Supporting Information). The extent of albuminuria, as assessed by the albumin‐creatinine ratio of spot‐collected urine samples every week after 25‐HC treatment and by 24‐hour urine albumin excretion at 18 weeks of age, was significantly attenuated in 25‐HC treated diabetic mice compared to vehicle‐treated diabetic mice (**Figure** **3**a,b). Plasma cholesterol levels were similar among groups (Figure 3c). Histologic analysis revealed that diabetes‐induced glomerular hypertrophy and mesangial expansion along with endothelial cell and podocyte loss were attenuated by 25‐HC treatment (Figure 3d–g). In addition, the 25‐HC treatment also reduced the infiltration of macrophages in the diabetic kidney (Figure S1, Supporting Information). Together, the above in vivo data demonstrate the protective effect of 25‐HC treatment in DKD. The above genetic and pharmacological studies suggest that CH25H and its product 25‐HC confer renoprotective effects in diabetic mice.

### 2.4 ARF4 is a target protein of 25‐HC in kidney Endothelial Cells

**Figure 4:**
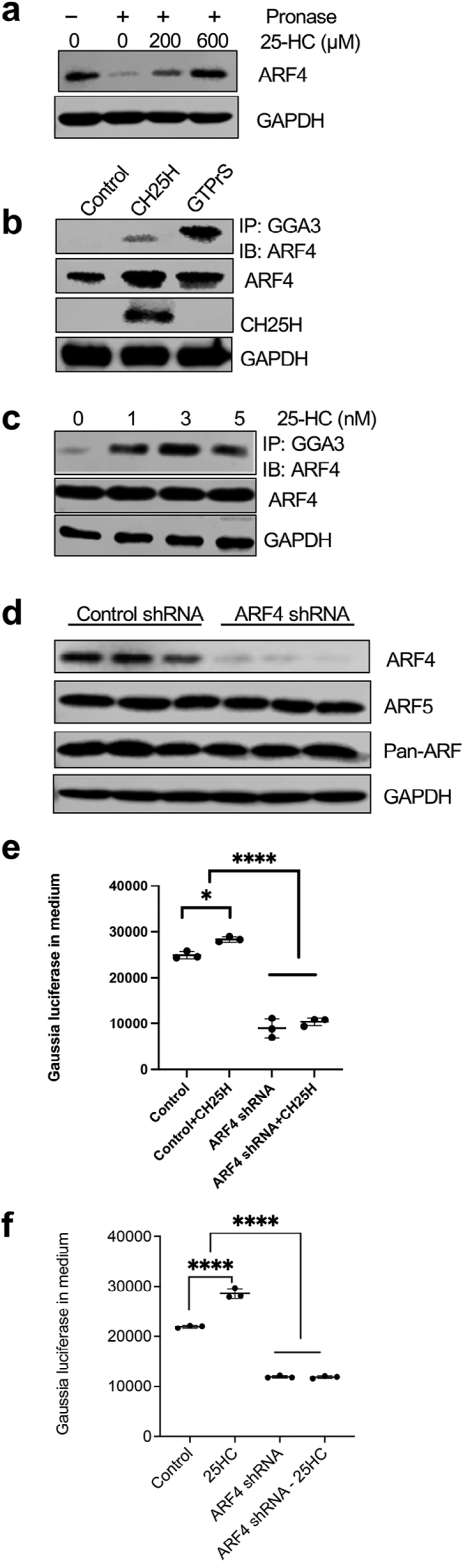
25‐HC binds to ARF4 to increase its activity. a) Western blot analysis of ARF4 after DARTS assay with different 25‐HC treatment concentrations. b) Lysates of HUVECs overexpressing CH25H were immunoprecipitated with GGA3 beads and immunoblotted for ARF4. The top panel shows the western blot immunoprecipitated protein and the bottom panel shows the total input. c) Lysates of HUVECs treated with different concentrations of 25‐HC were immunoprecipitated with GGA3 beads and immunoblotted for ARF4. The top panel shows the western blot immunoprecipitated protein and the bottom panel shows the total input. d) HUVECs were transduced with pLKO lentiviral control shRNA or shRNA against ARF4 (ARF4 shRNA). Lysates were subjected to western blot analysis using primary antibodies as indicated. e) HUVECs were transduced with pLKO lentiviral control shRNA or shRNA against ARF4 (ARF4 shRNA), as well as overexpressed with control vector or CH25H. HUVECs were transfected with Gaussia luciferase and Gaussia luciferase activity in the medium was measured. f) HUVECs were transduced with pLKO lentiviral control shRNA or shRNA against ARF4 (ARF4 shRNA), and treated with or without 25‐HC. HUVECs were transfected with Gaussia luciferase and Gaussia luciferase activity in the medium was measured.*P < 0.05, ****P < 0.0001 by 2‐way ANOVA.

Next, we determined the molecular mechanisms mediating the renoprotective effects of 25‐HC in DKD. First, we utilized the drug affinity responsive target stability (DARTS) method ^[^ ^18^ ^]^ to identify potential 25‐HC binding proteins. DARTS assay is based on the principle that binding of a certain molecule compound to its target proteins results in protein conformational changes when subjected to protease‐mediated digestion.^[^ ^18b,c^ ^]^ Mass spectrometry analysis was performed following the DARTS assay to reveal the potential 25HC‐interacting proteins in human umbilical vein endothelial cell (HUVEC) lysates incubated with vehicle or 25‐HC. ARF4 was at the top of the list of 25‐HC interacting proteins (**Table** **1**). We validated the interaction between 25‐HC and ARF4 by DARTS assay followed by western blot analysis (**Figure** **4**a; Figure S2, Supporting Information). It should be noted that the DARTS assay requires a high concentration of small molecules to identify its binding partners in cell lysates, which allows the determination of specific interaction qualitatively between 25‐HC and ARF4 but it is not sensitive enough to quantitatively assess the affinity of the interaction.

**Table 1:** Mass spectrometry analysis of 25‐HC interactive proteins.

Identified protein	Accession number	Molecular weight [kDa]	Spectra counts (EA)	Spectra counts (HC)	Ratio (HC/EA)	Normalized ratio (HC/EA)
ADP‐ribosylation factor 4 (ARF4)	P18085	21	0	8	5.0	5.6
Keratin, type II cytoskeletal 1 (KRT1)	P04264	66	2	16	4.5	5.0
Ras‐related protein Rab‐11B (RAB11B)	Q15907	24	0	5	3.5	3.9
Pre‐mRNA‐processing‐splicing factor 8 (PRPF8)	Q6P2Q9	274	0	5	3.5	3.9
Poly(rC)‐binding protein 3 (PCBP3)	P57721	39	0	5	3.5	3.9
60S ribosomal export protein NMD3 (NMD3)	Q96D46	58	0	4	3.0	3.3
Lipopolysaccharide‐responsive and beige‐like anchor protein (LRBA)	P50851	319	1	7	3.0	3.3
Brefeldin A‐inhibited guanine nucleotide‐exchange protein 1 (ARFGEF1)	Q9Y6D6	209	0	4	3.0	3.3
Sequestosome‐1 (SQSTM1)	Q13501	48	0	3	2.5	2.8
Nucleoporin NUP188 homolog (NUP188)	Q5SRE5	196	0	3	2.5	2.8

ARF4 is a small GTP binding protein whose active form is ARF4‐GTP.  Golgi‐localizing, gamma‐adaptin ear homology domain 3 (GGA3) is an ARF protein effector, which specifically binds to ARF‐GTP, but binds to inactive form (ARF‐GDP) with low affinity.^[^ ^15^ ^]^ We performed an immunoprecipitation assay with GGA3‐beads followed by western blots to detect active ARF4 in cells in control and HUVECs overexpressing CH25H (Figure 4b). The activity of ARF4 was enhanced by CH25H overexpression in comparison to control vector‐expressing HUVECs (Control), and G‐protein‐activating analog GTPrS‐treated cells served as positive control (Figure 4b). A dose‐dependent effect of 25‐HC on ARF4 activity was also observed (Figure 4c), confirming the above findings. As ARF4's function is essential for the regulation of the protein transport from the Golgi apparatus,^[^ ^15^ ^]^ we next examined the effects of ARF4 knockdown in Golgi transport in HUVECs. First, we confirmed the specific shRNA‐mediated ARF4 knockdown, as ARF5 or total ARF expression was not significantly changed (Figure 4d). We then utilized the *Gaussia*‐luciferase reporter,^[^ ^19^ ^]^ a 20 kDa protein that is actively secreted by cells, as a readout for functionality of the Golgi apparatus in HUVECs with or without CH25H overexpression and/or ARF4 knockdown. Indeed, CH25H overexpression increased the production of 25‐HC (15 ng mg^−1^ of protein), as measured by mass spectrometry (Figure S3, Supporting Information), and in secreted *Gaussia*‐luciferase activity in HUVECs (Figure 4e). However, this was abolished by ARF4 knockdown (Figure 4e). In addition, treatment of HUVECs with 25‐HC also increased *Gaussia*‐luciferase activity (Figure 4f). Together, these results confirmed that ARF4 is a target protein of 25‐HC and 25‐HC can regulate Golgi function via activation of ARF4 in endothelial cells.

### 2.5 25‐HC restored ARF4 activity by Blocking its interaction with ASAP1 in diabetic Endothelial Cells

**Figure 5:**
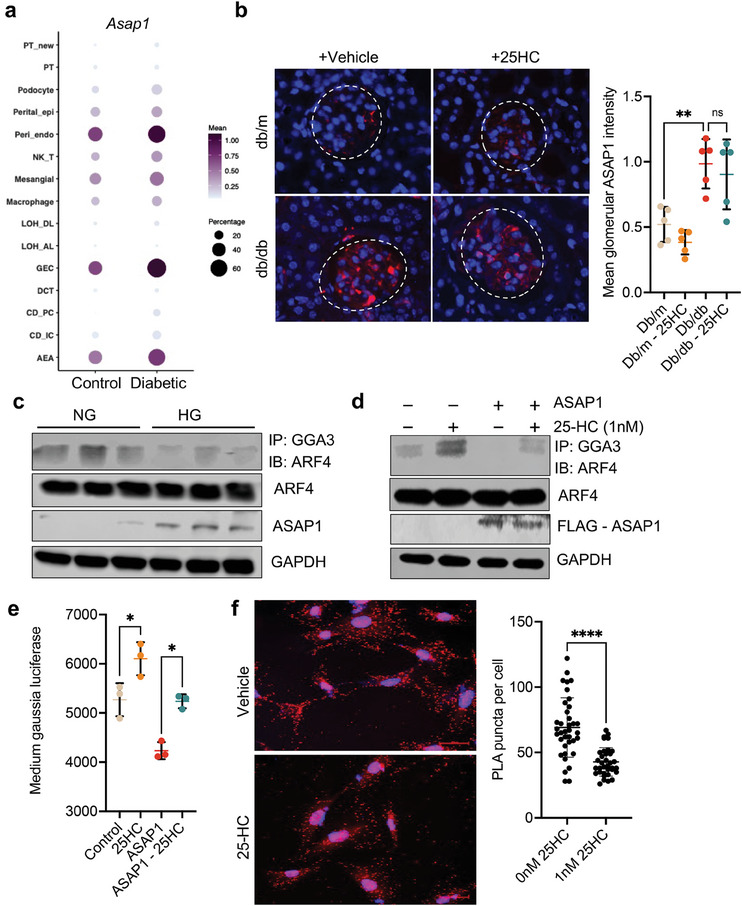
ASAP1 is increased in endothelial cells of diabetic mice and negatively regulates ARF4 activity. a) ASAP1 expression in mice kidney cells by single‐cell sequencing analysis of kidneys from diabetic OVE26 mice and control mice. b) Representative ASAP1 immunofluorescence images and quantification of mean glomerular ASAP1 intensity per mouse (*n* = 5 mice per group, 20–30 glomeruli scored per mouse). Scale bar = 20 µm. c) HUVECs were treated with normal glucose (NG) or high glucose (HG) and were immunoprecipitated with GGA3 beads and immunoblotted for ARF4. The top panel shows the immunoprecipitated proteins and the bottom panel shows total input.  d) HUVECs were infected with Flag‐tagged ASAP1 or control vectors and then treated with either vehicle or 1 nm 25‐HC. Cells were immunoprecipitated with GGA3 beads and immunoblotted for ARF4. The top panel shows the immunoprecipitated proteins and the bottom panel shows total input. e) Measurement of Gaussia luciferase activity in the medium of HUVECs overexpressed with or without ASAP1 and treated with or without 25‐HC. f) Representative images and quantification of proximity ligation assay (PLA) between ASAP1 and ARF4 in HUVECs treated with or without 25‐HC. *P < 0.05, **P < 0.01 by 2‐way ANOVA. ****P < 0.0001 by *t*‐test. ns, non‐significant.

To better understand the regulation of ARF4 activity, we analyzed our recent scRNA‐seq dataset of murine diabetic kidneys to examine endothelial cell‐specific differentially expressed genes, which are involved in the regulation of ARF4. We found that ARFGAP with SH3 domain, ankyrin repeat, and PH domain1 (ASAP1), expressed highly in endothelial cells including GECs and PTCs, and their expression increased in diabetic mice (**Figure** **5**a). Immunofluorescence staining confirmed that expression of ASAP1 was significantly increased in Lepr^db/db^ diabetes mice compared to their controls (Figure 5b). Consistently, high glucose treatment of HUVECs increased the expression of ASAP1 and in the reduction of active form of ARF4 (Figure 5c). These results suggest that an increase of ASAP1 expression leads to a decrease of ARF4 activity in endothelial cells of diabetic kidneys. Next, we examined whether 25‐HC was able to restore ARF4 activity by interrupting the interaction between ARF4 and ASAP1. We showed that overexpression of ASAP1 reduced ARF4 activity in HUVECs, but 25‐HC treatment was able to restore the activity of ARF4 in ASAP1 overexpressing cells (Figure 5d). Moreover, *Gaussia*‐luciferase activity was restored by 25‐HC treatment in ASAP1 overexpressing HUVECs (Figure 5e) and proximity ligation assays further confirmed that 25‐HC treatment was able to interrupt the interaction between ARF4 and ASAP1 (Figure 5f). Together, these data suggest that in diabetic kidneys, expression of ASAP1, an ARF4‐GAP, increases in glomerular and peri‐tubular endothelial cells, leading to reduced ARF4 activity and Golgi malfunction. 25‐HC helps to restore ARF4 activity and Golgi function in endothelial cells of diabetic kidneys, in part through inhibiting the interaction between ASAP1 and ARF4, to confer renoprotection in DKD.

### 2.6 CH25H Attenuates diabetes‐induced Endothelial Cell injury by Activating ARF4

**Figure 6:**
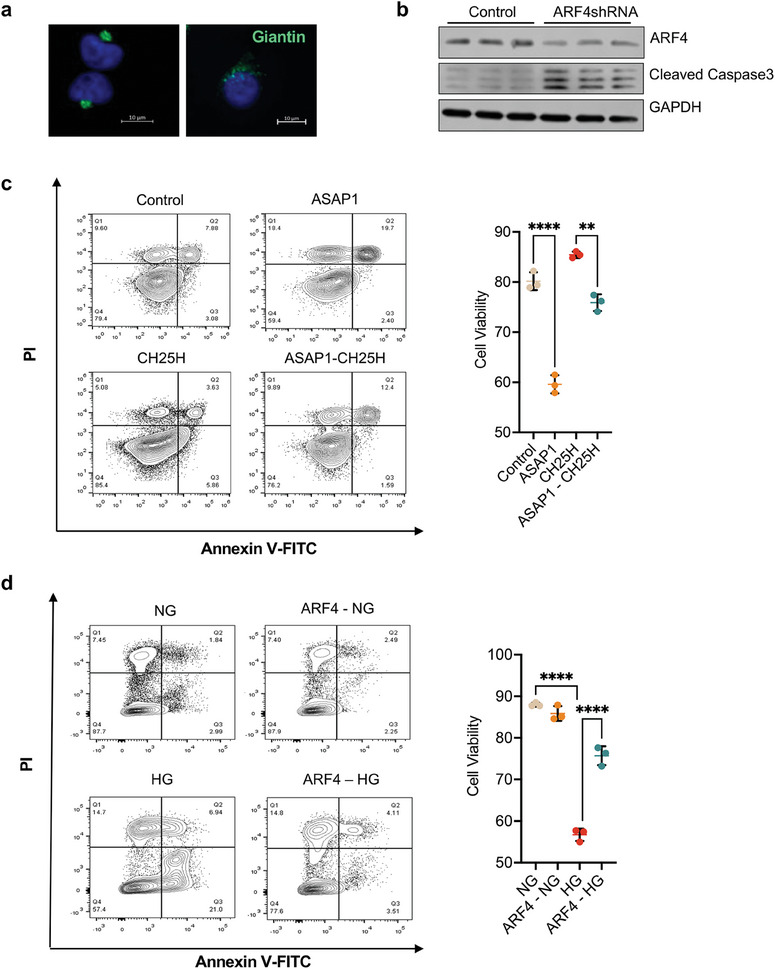
CH25H protects HUVECs from cell death by increasing ARF4 activity. a) HUVECs were transduced with pLKO lentiviral control shRNA or shRNA against ARF4 (ARF4 shRNA). Representative images of Giantin staining of HUVECs expressing control vector or shRNA against ARF4. Scale bar = 10 µm. b) HUVECs were transduced with pLKO lentiviral control shRNA or shRNA against ARF4 (ARF4 shRNA). Lysates were subjected to western blot analysis using primary antibodies as indicated. c) Cell viability measurement of HUVECs overexpressed with control vector or ASAP1 or/and CH25H. d) Cell viability measurement of HUVECs overexpressed with control vector or ARF4 and treated with normal glucose (NG) or high glucose (HG). **P < 0.01, ****P < 0.0001 by 2‐way ANOVA.

To investigate the effect of ARF4 on endothelial cell function, we further examined the effects of ARF4 knockdown in cultured HUVECs. Consistent with the above findings of reduced Golgi transport of *Gaussia*‐luciferase reporter, reduction of ARF4 resulted in the dispersal of Golgi apparatus, as assessed by Giantin‐stained area (**Figure** **6**a; Figure S4, Supporting Information). This was associated with increased cleaved caspase 3 (Figure 6b), suggesting that ARF4 insufficiency promotes Golgi dysfunction and subsequent endothelial cell apoptosis. Since ASAP1 is upregulated in endothelial cells of diabetic kidneys, we overexpressed ASAP1 in HUVECs to mimic diabetic conditions and performed flow cytometry to assess cell viability. We found that ASAP1 overexpression increased cell death, which was partially rescued by CH25H overexpression (Figure 6c). We then overexpressed ARF4 in HUVECs (Figure S5, Supporting Information), which attenuated cell death induced by high glucose treatment (Figure 6d). Together, these data suggest that increased ASAP1 in diabetic conditions leads to suppression of ARF4 activity, Golgi dysfunction, and cell death, which could be rescued by activation of the CH25H‐25HC pathway.

### 2.7 25‐HC does not affect Podocyte Apoptosis

25‐HC is a small molecule and much more hydrophilic than cholesterol itself. Therefore, it is likely that 25‐HC could cross the glomerular barrier and directly exert its effects on podocytes via the paracrine mechanism. To test this, we treated cultured human podocytes with 25‐HC under normal or high glucose conditions for 24 h. We found that 25‐HC did not affect podocyte apoptosis as assessed by TUNEL staining (Figure S6, Supporting Information). Since 25‐HC is known to regulate sterol regulatory element‐binding protein (SREBP) expression, and lipid metabolic disorder plays a major role in podocyte injury in DKD,^[^ ^20^ ^]^ we also examined SREBP1 expression in podocytes but we found that treatment of 25‐HC did not change SREBP1 expression in podocytes (Figure S7, Supporting Information).

## 3 Discussion

Our study demonstrates that CH25H, an endothelial cell‐specific gene that was upregulated in both mouse and human diabetic kidneys, is protective against diabetic kidney diseases, which could be a compensatory mechanism to prevent DKD progression. Therefore, diabetic patients with high CH25H/25‐HC levels may have slower DKD progression than those with low CH25H/25‐HC levels. Indeed, the lack of CH25H led to worsened DKD progression in diabetic mice. Recently, we identified several genes, that are upregulated in early DKD, and suppression of these genes led to worsened progression of kidney disease.^[^ ^21^ ^]^ However, this needs to be further confirmed in future studies. Future studies are also required to determine whether expression levels of CH25H in early DKD could predict the disease progression.

However, diabetic mice with even elevated CH25H nevertheless developed DKD. This suggests that albeit protective, moderately increased endogenous CH25H in early diabetic kidneys may not be sufficient compensation to prevent DKD development. This notion is supported by the fact that exogenous administration of 25‐HC to diabetic mice further protects against DKD. An alternative way to increase 25‐HC is to block cytochrome P450 family 7 subfamily B member 1 (Cyp7b1), which metabolizes 25‐HC to 7*α*, 25‐HC. Levels of 25‐HC in plasma, liver, and kidney were significantly increased in *Cyp7b1* knockout mice without affecting plasma cholesterol, tissue cholesterol, and intestinal cholesterol absorption.^[^ ^22^ ^]^ The role of Cyp7b1 in DKD has not been studied yet.

We sought to examine the role of CH25H further in this study, as its expression was largely limited to endothelial cells in the kidney and upregulated in early DKD. Although CH25H has been reported to have diverse biological functions, its role in renal endothelial cell biology and pathology has never been investigated. Our in vivo studies showed that the lack of CH25H led to apoptosis of GECs and peri‐tubular endothelial cells, indicating that CH25H has a pro‐survival effect on endothelial cells in the context of diabetes. Kidney vascular endothelial cell dysfunction is one of the pathological hallmarks of DKD, which is an emerging attractive intervention target to treat DKD. The vascular endothelial cell dysfunction in DKD is multifaceted.  Altered nitrogen oxide (NO) production, reactive oxygen species production, and lipid metabolism are involved in endothelial cell injury in DKD.^[^ ^23^ ^]^ It is known that GECs undergo neo‐angiogenesis at the early stage but their apoptosis increases in parallel leading to decreased GECs in progressive DKD.^[^ ^5^ ^]^ We found that CH25H loss caused significant kidney endothelial cell apoptosis in diabetic mice, which may underlie the more severe DKD phenotype in the diabetic Ch25h‐null mice. Thus, our findings highlight the importance of CH25H in maintaining the viability of endothelial cells under diabetic conditions.

We were also interested in further examining CH25H's role in DKD because it is involved in lipid metabolism. The best‐known function of CH25H and its product 25‐HC is to regulate cholesterol metabolism by inhibiting the activation of SREBPs that activate gene expression related to lipid synthesis.^[^ ^24^ ^]^ Although SREBPs are elevated in diabetic kidneys,^[^ ^25^ ^]^ the endothelial cell apoptosis in Ch25h*^−/−^* diabetic kidneys cannot be attributed to SREBP dysregulation, as SREBP‐1 overexpression in kidneys did not induce cell apoptosis in non‐diabetic or STZ‐induced diabetic mice.^[^ ^26^ ^]^ Moreover, Ch25h homozygous knockout mice have normal lipid metabolism. Lipid metabolic disorder has been reported to play a major role in podocyte injury in DKD.^[^ ^20^ ^]^ However, we did not find any effect of 25‐HC on SREBP1 expression in podocytes. Therefore, we searched for 25‐HC interactive proteins other than SREBPs.

Using DARTS and mass spectrometry approach, we identified ARF4, a small GTP binding protein involved in Golgi apparatus activities, interacting with 25HC. ARF4 conventional knockout mice did not show kidney phenotype,^[^ ^27^ ^]^ indicating that ARF4 is dispensable for mouse kidney development and functions without extra stresses. However, our data suggest that ARF4 has a significant renoprotective effect in the context of diabetes. To understand the mechanisms mediating the effects of 25‐HC and ARF4 in endothelial cells, we performed a series of in vitro studies in HUVECs. First, we provided evidence that ARF4 activity was regulated by ASAP1, a GAP protein for ARF4. Interestingly, ASAP1 is predominantly expressed in endothelial cells and upregulated in diabetic kidneys, which may contribute to the low ARF4 activity in endothelial cells of diabetic kidneys. We showed that 25‐HC was able to interrupt ASAP1‐ARF4 interaction, thereby increasing ARF4 activity and preventing cells from apoptosis in the context of diabetes.

Taken together, the significant endothelial cell apoptosis in diabetic *Ch25h‐*null mice is likely attributed to the loss of ARF4 activity in the Golgi apparatus. Under diabetic conditions, ASAP1 in endothelial cells was upregulated leading to low ARF4 activity. In addition, the inhibitory effects on ARF4‐ASAP1 interaction are lost due to low or no production of 25‐HC, leading to more inhibition of ARF4 activity by ASAP1. Therefore, the activities of ARF4 were further inhibited in endothelial cells with both ASAP1 expressing and *Ch25h* deletion.

Our work thus highlights the potential role of ASAP1 in the development of DKD. ASAP1 is upregulated in diabetic mouse and human kidneys. Interestingly, a single nucleotide polymorphism (SNP) of ASAP1 was identified to be associated with DKD in diabetic patients of Mexican ancestry.^[^ ^28^ ^]^ The SNP is located in an intron of ASAP1 and the role of the SNP in the regulation of ASAP1 expression has not yet been examined. It is worth noting that ASAP1 not only regulates ARF4 but also (even with greater activity toward) ARF1 and ARF5. It is speculated that higher levels of ASAP1 could greatly affect the activities of the Golgi apparatus by deactivating a broad spectrum of ARF proteins. In addition to deactivating ARF proteins, ASAP1 also regulates the actin cytoskeleton by binding to the regulators of the actin cytoskeleton, such as the tyrosine kinase Src and phosphatidylinositol 4,5‐bisphosphate.^[^ ^29^ ^]^ As GECs are fenestrated endothelial cells, remodeling of their cytoskeletal structures by ASAP1 may also affect the morphology and permeability of GECs.

By using DARTS and Mass Spectrometry analysis, we also observed that 25‐HC interacts with Rab11b, another important small G protein that is involved in regulating vesicular trafficking through the recycling of endosomal compartment and early endosomes to the trans‐Golgi network and plasma membrane.^[^ ^30^ ^]^ Future studies are required to determine whether 25‐HC could affect Golgi function via regulation of Rab11b.

We also found a significant podocyte loss in diabetic *Ch25h* knockout mice. This is likely due to the GEC‐podocyte crosstalk, as GEC injuries can lead to podocyte injuries.^[^ ^31^ ^]^ Apoptotic GECs in diabetic *Ch25h* knockout mice inevitably would decrease the production of NO, which would result in podocyte damage. Another possibility is that 25‐HC, a much more hydrophilic compound than cholesterol itself, could cross the glomerular barrier and directly exert its protective functions in podocytes. However, our scRNAseq data suggest that podocytes express a relatively low level of ASAP1, and we could not detect the expression of ASAP1 in podocytes by staining of kidneys. In addition, we confirmed that treatment of podocytes with 25‐HC did not rescue high glucose‐induced podocyte apoptosis. Therefore, the protective effect of 25‐HC in podocytes is likely indirect from GEC‐podocyte crosstalk.

CH25H/25‐HC is also known to regulate inflammatory pathways. For instance, studies have shown that activation of macrophages lacking CH25H expression leads to overproduction of inflammatory interleukin‐1 family cytokines ^[^ ^32^ ^]^ and that CH25H reduces lipopolysaccharide (LPS)‐induced macrophage TNF‐*α* expression and secretion,^[^ ^33^ ^]^ indicating an anti‐inflammatory effect of 25‐HC. But 25‐HC has also been shown to enhance polyinosinic: polycytidylic acid‐induced macrophage IL‐6 production,^[^ ^34^ ^]^ suggesting a pro‐inflammatory role. Therefore, the role of CH25H/25‐HC in the regulation of inflammation is controversial and may also have tissue and disease‐specificity. We showed here that mice treated with 25‐HC had reduced infiltration of macrophages. However, this could result from either direct or indirect effects of 25‐HC.

Our studies have several limitations. We have not generated endothelial cell‐specific *Ch25h* knockout mice to study the role of CH25H specifically in endothelial cells. The roles of ARF4 and ASAP1 in DKD have not been studied in vivo by using conditional ARF4 and ASAP1 knockout mice. Though we demonstrated that *Ch25h* deletion or 25‐HC treatment did not affect plasma cholesterol levels in mice, whether plasma cholesterol regulates CH25H expression and 25‐HC production in endothelial cells remains to be determined. Although our data suggest that Golgi dysfunction likely contributes to endothelial cell injury in DKD, whether Golgi dysfunction also contributes to other kidney abnormalities in DKD such as kidney hypertrophy and tubular cell injury is not yet known.

**Figure 7:**
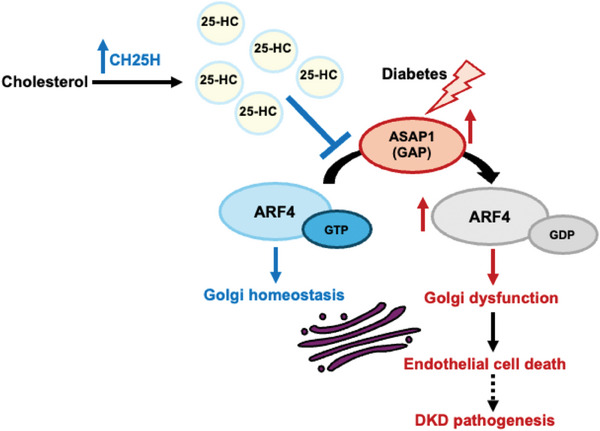
Protective effects of CH25H/25H in DKD. CH25H/25HC protects diabetic kidneys by maintaining ARF4 activity via inhibiting ARF4 interaction with its GAP, ASAP1. Increased ASAP1 in diabetic kidneys leads to Golgi and endothelial cell dysfunction to promote DKD.

In summary, the present study supports the critical role of *Ch25h* and its product 25‐HC in protecting against diabetes‐induced kidney endothelial cell injury (**Figure** **7**). Mechanistically, 25HC enhances the activity of ARF4 by interrupting ARF4‐ASAP1 interaction. Maintaining high ARF4 activity could help to keep endothelial cell survival by maintaining normal Golgi function in the diabetic condition. This is particularly important because ARF4 activity is low in the diabetic kidney due to a high expression of ASAP1. Our work suggests that dysfunction of the Golgi apparatus could be also involved in the pathogenesis of DKD, which has not been studied in the past. Our studies also suggest that 25‐HC could be potentially developed as a new therapy against endothelial cell injury in DKD but also for other diabetic complications.

## 4 Experimental Section

### Reagents and Antibodies 

25‐hydroxycholesterol (25‐HC) was purchased from Cayman Chemical (Cat. # 11 097). Antibodies of anti‐CH25H antibody (Abcam ab133933), anti‐GAPDH antibody (Cell Signaling Technology Cat. 2118), anti‐Giantin (ab80864), anti‐WT1 antibody (Abcam, ab89901), anti‐CD31 antibody (BD Biosciences, 550 274), anti‐ARF4 (Abcam ab171746,  PA5‐37841), anti‐ARF5 (Avnova, H00000381‐M01), anti‐Pan‐ARF (Invitrogen, MA3‐060), anti‐cleaved caspase 3 (Cell Signaling Technology Cat. 9661), anti‐HA (Abcam ab236632), anti‐FLAG (Sigma F3165), anti‐ASAP1 (Bethyl A302‐118A), anti‐SREBP1 (NOVUS, NB600‐582SS) antibodies were purchased commercially.

### Cell Culture 

HUVECs were cultured in an endothelial cell basal medium (PromoCell c‐22220). Retroviral temperature‐sensitive SV40 immortalized human podocytes were cultured in RPMI1640 medium with 10% fetal bovine serum and 1% ITS at 33 °C for growth. Podocytes were transferred to 37 °C for at least 3 days for differentiation before use.

### Western Blot

Cells were lysed in M‐PER mammalian protein extraction reagent (Thermo Fisher, 78 502) containing a protease inhibitor mixture. The cell lysate was then separated on SDS‐PAGE and transferred to the nitrocellulose membrane. The membrane was subjected to immunoblot analysis with specific antibodies.

### Mouse Model

*Ch25h* ^−/‐^ mice on a C57BL/6J genetic background (Jackson lab Stock No: 01 6263) and heterozygous *Lepr^db^* ^/+^ on a C57BL/6J genetic background (Jackson lab Stock No: 000697) were purchased from Jackson Lab. *Ch25h* ^−/−^ mice were first mated with Lepr*^db^* ^/m^ mice to generate *Ch25h* ^−/+^/ Lepr*^db^* ^/m^ mice. Littermates male and female *ch25h* ^−/+^/ Lepr*^db^* ^/+^ mice were next crossed to generate 4 groups of mice: *Ch25h* ^+/+^/Lepr^db/m^, *Ch25h* ^−/−^/Lepr^db/m^, *Ch25h* ^+/+^/Lepr^db/db^ and *Ch25h* ^−/−^/Lepr^db/db^). For the 25‐HC treatment study, 10 weeks of male Lepr^db/m^ and Lepr^db/db^ mice on C57BLKS/J genetic background were purchased from Jackson Lab (stock # 000642). 25HC dissolved in 5% ethanol or vehicle (5% ethanol) was given to Lepr^db/m^ and Lepr^db/db^ mice by tail vein injection at a dose of 0.16 mg k^−1 ^g body weight, once a day, 5 days a week for 8 weeks, as previously described.^[^ ^35^ ^]^ Urine albumin was quantified by ELISA using a kit from Bethyl Laboratories, Inc. (#E99‐134). Urine creatinine levels were measured in the same samples using the QuantiChrom creatinine assay kit (DICT‐500, BioAssay Systems) according to the manufacturer's instructions. The urine albumin excretion rate was expressed as the ratio of albumin to creatinine. Twelve‐hour urine collections in the metabolic cages were also used for the determination of urinary albumin excretion.

### Kidney Histology

Mouse kidneys were perfused and fixed using freshly prepared formaldehyde (4% in PBS), embedded in paraffin or cryopreserved as frozen samples, and processed as 4‐micron sections. Deparaffinized kidney sections were stained with periodic acid–Schiff solution. Assessment of the mesangial and glomerular cross‐sectional areas was performed by pixel counts on 20–30 glomeruli per section under 400× magnification by the operator blinded to the experimental group.

### Immunofluorescence Staining

Paraffin‐embedded kidney sections of 4 µm thickness were deparaffinized and blocked in PBS with 2% goat serum and 2% bovine serum albumin for 30 min. Sections were then incubated with primary antibodies at 1:50 overnight at 4 °C. After 3 washes with PBS, slices were incubated with fluorescence‐labeled secondary antibodies (Invitrogen) for 1 h at 1:200. Slices were finally mounted and observed under a microscope (Zeiss A×10 microscope). Fluorescence intensity was measured with ImageJ.

### In Situ Hybridization

RNAscope in situ hybridization was performed on paraffin‐embedded kidney sections as instructed by advanced cell diagnostics (ACD). Probes for mouse *Ch25h* and CD31 were purchased from ACD (Cat No. 424 561 and  316721‐C2, respectively). RNAscope in situ hybridization for human CH25H was performed using a probe mixture. Sequences of three probes were as follows: AGT TCC TGC AGC CCC TCT GGG ACC ACC TGA GGA GCT G; ACC ACT CCG GCT ACA ACT TCC CTT GGT CCA CTC ACA GAC T and ACT GCA ACT TCG CTC CGT ACT TTA CAC ACT GGG ACA AAA T.

### DARTS

DARTS and mass spectrometry analysis were performed as previously described.^[^ ^18^ ^]^ Briefly, HUVEC lysates in M‐PER mammalian protein extraction buffer (Thermo Fisher 78 501) with proteinase inhibitor (Roche, 11 836 153 001) were incubated with 25HC or vehicle for 1 h at room temperature. Lysate was then digested with pronase (Roche, 10 165 921 001) at a ratio of 1:1000 for 20 min at room temperature. Protein samples were subject to mass spectrometry analysis and western blot analysis.

### Mass Spectrometry Analysis

25‐HC interactive proteins in DARTS were analyzed at the Center for Advanced Proteomics Research at the New Jersey Medical School (NJMS). 25‐HC in cell lysate was analyzed at UCSD Lipidomics Core.

### Plasmid and Transfection

Human ARF4 plasmid was purchased from Dharmacon (clone # 2 900 495). ARF4 was amplified with a forward primer containing a BamH1 cleavage site and a reverse primer with an Xmal cutting site incorporated with the FLAG sequence. The forward primer sequence was GGA TCC ATG GGC CTC ACT ATC TCC TC and the reverse primer sequence is CTC GAG TTA CTT GTC GTC ATC GTC TTT GTA GTC ACG TTT TGA AAG CTC. DNA sequences were validated by Sanger DNA sequencing. Human CH25H‐HA (EX‐Q0427‐Lv117) and ASAP1‐FLAG (EX‐H0193‐Lv101) were purchased from Genecopoeia. Gaussia lueciferase (N8081S) plasmid was purchased from New England Biolabs. GGA3‐FLAG plasmid was purchased from Addgene (11 185). Empty pLKO.1 shRNA (SHC001) and human ARF4 shRNA (TRCN0000286251) plasmids were purchased from Sigma.

### Transfection and Lentiviral Preparation

Human wildtype ARF4 expression plasmids or shRNA plasmids together with packaging and envelope plasmids were transfected into 293 Lenti cells with Polyjet transfection reagent (Signagen SL100688). Human CH25H and ASAP1 viral particles were made with a Lenti‐Pac HIV expression packaging kit (Genecopoeia LT001). The cell medium was collected per day for 3 days. The medium was centrifuged at 500 g for 5 min to get rid of cell debris. The medium was then centrifuged at 5000 g for 4 h at 4 °C. Supernatant was removed, and the virus pallet was dissolved in serum‐free endothelial cell basal medium (PromoCell C‐22220). Gaussia luciferase and GGA3 plasmid were transfected into HUVECs with Cytofect HUVEC Transfection kit (TF200K).

### Terminal dUTP nick‐End Labeling (TUNEL) 

Frozen mouse kidney samples were cut into 4µm thick sections. The sections were treated with the DeadEnd Fluorometric TUNEL system (Promega, G3250) following the manufacturer's instructions. Sections were mounted with antifade mounting medium with DAPI (Vector Laboratories, H‐1500‐10) and visualized on a Zeiss A×10 microscope.

### Apoptosis by flow Cytometry

ARF4 knockdown and control cells were harvested and incubated with Annexin and Propidium iodide in 1× binding buffer for 30 min. Fluorescence was measured by flow cytometry (FACSAria; BD Biosciences). Data were analyzed by Flowjo software.

### Proximity Ligation Assay

PLA was performed using Duolink In Situ PLA Probe anti‐Goat PLUS  (DUO92003) and anti‐Rabbit‐Minus (DUO92005) following the manufacturer's instructions.

### Statistics

Data were presented as mean ± SD. The unpaired *t*‐test was used for comparisons between two groups and two‐way ANOVA followed by Tukey's post hoc analysis was used for comparisons between multiple groups using the GraphPad Prism software. *P* < 0.05 was considered statistically significant.

## Conflict of Interest

The authors declare no conflict of interest.

## Author Contributions

L.Z., Z.F., Q.Z., and S.Y. have contributed equally to this work. J.C.H., R.L., Y.Z., and K.L. designed the research project. L.Z., Z.F., Q.Z., S.Y., J.F., Z.S., G.L., C.W., and Z.Z. performed the experiments. L.Z., Z.F., Q.Z., S.Y., and K.L. analyzed the data. J.C.H., R.L., K.L., and Y.Z. drafted and revised the manuscript. All authors approved the final version of the manuscript.

## Supporting information

Supporting Information

## Data Availability

The data that support the findings of this study are available from the corresponding author upon reasonable request.
