# Supplementary material for: Cholesterol 25‐Hydroxylase Protects Against Diabetic Kidney Disease by Regulating ADP Ribosylation Factor 4
Source: Adv Sci (Weinh). 2024 May 30;11(29):2309642. doi: 10.1002/advs.202309642 (PMC11304234; doi:10.1002/advs.202309642)
Supplement: Supplementary file 1 — Supporting Information [file ADVS-11-2309642-s001.docx]

**Supplementary Materials**

Table S1. Body weight and blood glucose of Lepr^db/m^ and Lepr^db/db^ mice treated for 8 weeks with vehicle or 25-HC.

|  | **Body Weight (g)** | | | | | **Blood glucose (mg/dL)** | | | | |
| --- | --- | --- | --- | --- | --- | --- | --- | --- | --- | --- |
|  | **#1** | **#2** | **#3** | **#4** | **#5** | **#1** | **#2** | **#3** | **#4** | **#5** |
| *db/m* | 28.4 | 28.6 | 28.6 | 28.2 | 27.9 | 116.4 | 126.6 | 108.3 | 136.5 | 129.4 |
| *db/m* + 25HC | 30.5 | 28.5 | 29.2 | 29 | 28.3 | 116.4 | 130.6 | 106.7 | 116.5 | 126.4 |
| *db/db* | 51.5 | 52.4 | 54.8 | 51.8 | 49.5 | 401.3 | 365.1 | 394.6 | 445.2 | 325.4 |
| *db/db* + 25HC | 52.5 | 51.8 | 50.5 | 56.8 | 50.8 | 346.7 | 463.8 | 359.4 | 406.4 | 346.9 |


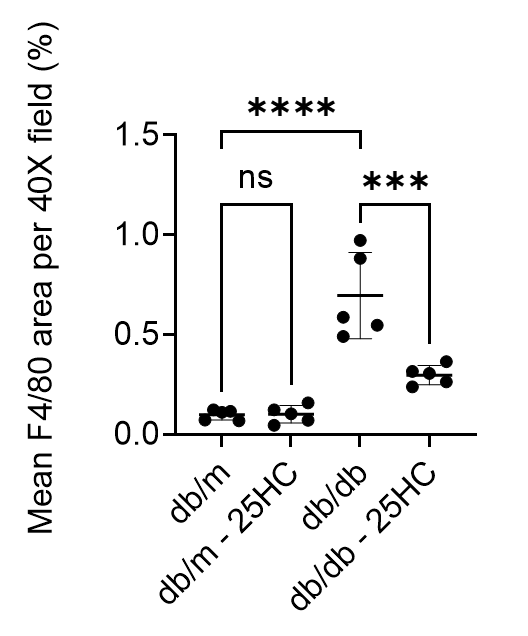


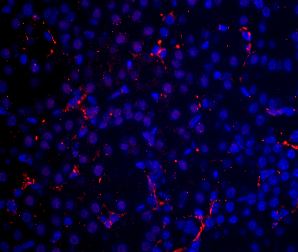

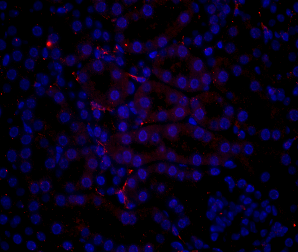

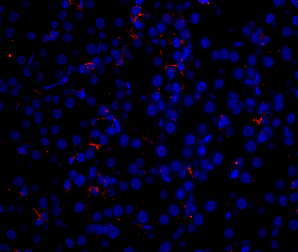

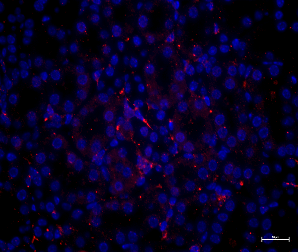


25HC

Control

db/m

db/db

**F4/80**

Fig. S1. Representative images and quantification of F4/80 immunofluorecence of control (db/m) and diabetic (db/db/) mice treated with or without 25HC. Scale bar = 10 μm. *** P < 0.001, **** P < 0.0001 by 2-way ANOVA.

Fig. S2. Quantification of western blot analysis of ARF4 after drug affinity responsive target stability (DARTS) assay with different 25-HC treatment concentration.

**b**

**a**

**Ch25h-HA**

**GAPDH**

**Control Vector**

**Ch25h overexpression**

Fig. S3. Ch25h overexpression increased 25-hydrocholesterol production in HUVECs. a. HA-tagged Ch25h was expressed in HUVECs by viral infection. b. 25-hydrocholesterol measurement by mass spectrometry.


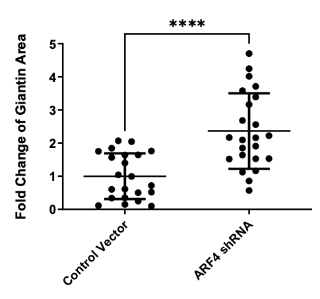


Fig. S4. Quantification of Giantin stained area in HUVECs tranduced with pLKO lentiviral control shRNA or shRNA against ARF4 (ARF4 shRNA).

**ARF4**

**GAPDH**

**GFP**

**ARF4 overexpression**

Fig. S5. Western blots showing ARF4 overexpression in HUVEC cells.


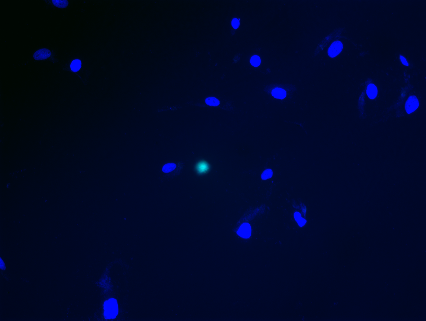

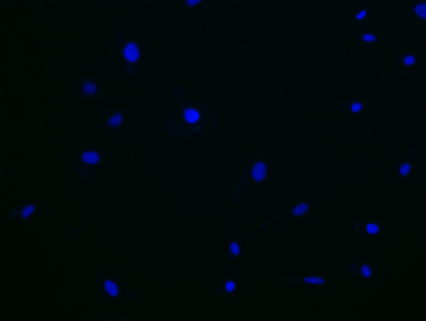

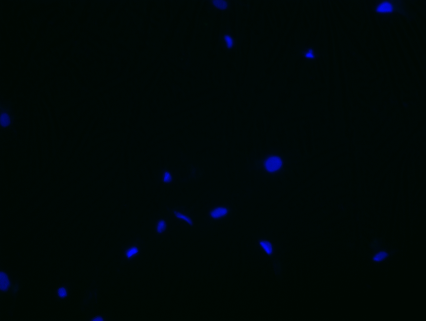

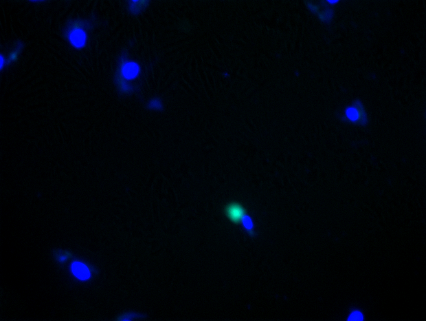


**TUNEL**

25HC

Control

NG

HG

Fig. S6. Representative images of TUNEL staining of podocytes treated with normal glucose (NG) or high glucose (HG), with or without 25HC treatment.

GAPDH

P-SREBP1

N-SREBP1

0 1 3 5 25HC (nM)

Fig. S7. Podocytes were treated with indicated concentration of 25HC. Lysates were subjected to western blot analysis using primary antibodies as indicated.
